# MAPK4 deletion enhances radiation effects and triggers synergistic lethality with simultaneous PARP1 inhibition in cervical cancer

**DOI:** 10.1186/s13046-020-01644-5

**Published:** 2020-07-25

**Authors:** Shuzhen Tian, Lili Lou, Mengyuan Tian, Guangping Lu, Jianghua Tian, Xi Chen

**Affiliations:** 1 Department of Gynecology, Henan Cancer Hospital, No. 127 Dongming Avenue, Zhengzhou City, 450009 Henan Province China; 2 Department of Respiratory Medicine, The First Affiliated Hospital Zhengzhou University, Zhengzhou City, 450052 Henan Province China; 3 Department of Pathology, Henan Cancer Hospital, Zhengzhou City, 450009 Henan Province China; 4 Department of Emergency Medicine, The First Affiliated Hospital Zhengzhou University, Zhengzhou City, 450052 Henan Province China; 5 Department of Internal Medicine, Peking University Hospital, Beijing, 100871 China; 6 School of Basic Medicine, Zhejiang University Medical School, Hangzhou City, 310013 Zhejiang Province China

**Keywords:** Cervical cancer, MAPK4, AKT phosphorylation, DNA double-chain breakage, Radiation sensitivity, PARP1 inhibitors

## Abstract

**Background:**

Cervical cancer is one of the most common cancers among females worldwide and advanced patients have extremely poor prognosis. However, adverse reactions and accumulating resistance to radiation therapy require further investigation.

**Methods:**

The expression levels of mitogen-activated protein kinase 4 (MAPK4) mRNA were analyzed by real-time PCR and its association with overall survival was analyzed using Kaplan-Mier method. Colony formation, immunofluorescence and western blotting were used to examine the effects of MAPK4 knockout or over-expression on cervical cancer cells after radiation treatment. Drug-sensitivity of cervical cancer cells to PARP1 inhibitors, olaparib or veliparib, was analyzed by CCK-8 cell viability assays, and the 50% inhibitory concentration (IC50) was quantified using GraphPad Prism. The functional effects of MAPK4 knockout on the sensitivity of cervical cancer to radiation treatment and PARP1 inhibitors were further examined using xenograft tumor mouse models in vivo.

**Results:**

Cervical cancer patients with high MAPK4 mRNA expression have lower survival rate. After radiation treatment, the colony number of MAPK4 knockout cells was markedly reduced, and the markers for DNA double-chain breakage were significantly up-regulated. In addition, MAPK4 knockout reduced protein kinase B (AKT) phosphorylation, whereas its over-expression resulted in opposite effects. In MAPK4 KO cells with irradiation treatment, inhibition of AKT phosphorylation promoted DNA double-chain breakage. Constitutive activation of AKT (CA-AKT) increased the levels of phosphorylated-AKT (p-AKT), and DNA repair-related proteins, phosphorylated-DNA-dependent protein kinase (p-DNA-PK) and RAD51 recombinase (RAD51). Furthermore, MAPK4 knockout was found to affect the sensitivity of cervical cancer cells to poly ADP-ribose polymerase 1 (PARP1) inhibitors by activating the phosphorylation of AKT. Moreover, in vivo results demonstrated that MAPK4 knockout enhanced the sensitivity of cervical cancer to radiation and PARP1 inhibitors in mouse xenograft models.

**Conclusions:**

Collectively, our data suggest that combined application of MAPK4 knockout and PARP1 inhibition can be used as therapeutic strategy in radiation treatment for advanced cervical carcinoma.

## Background

With over half a million cases occurring annually worldwide, cervical cancer remains the second most common cancer and third leading cause of cancer-related death among females in developing countries [1–3]. Although human papillomavirus (HPV) vaccines are available and are effective in reducing the incidence and mortality, improved treatment for cervical cancer remains an urgent need [4]. In particular, patients with advanced cervical cancer have extremely poor prognosis and low survival rate [5]. According to National Comprehensive Cancer Network (NCCN) 2016, radiotherapy can be performed in all stages of patients [6]. However, increased dose of radiation inevitably leads to adverse reactions, and accumulating resistance to radiation therapy needs to be taken into account [7]. Therefore, questions remain as to which factors contribute to the radioresistance and poor outcomes of cervical cancer.

As a family of evolutionarily conserved enzymes, (mitogen-activated protein kinase) MAP kinases play a critical role in signaling cascades, which mediate a wide range of extracellular stimuli and result in different cellular responses, including cell proliferation, cell motility, cell cycle, cytokine biosynthesis, and chromatin remodeling and so on [8]. A total of 13 human MAP kinases have been identified, including ERK1/2 (MAPK3/1), P38α/β/γ/δ (MAPK14/11/12/13) and JNK1/2/3 (MAPK8/9/10) [9]. MAPK4, also known as ERK4, p63 MAPK, ERK3β and Prkm4, has been mapped to chromosome 18q12–21 following the identification of ERK1, ERK2 and ERK3 [10]. As an atypical MAPK, MAPK4 lacks TXY and APE motifs, which are highly conserved in other MAPKs. Instead, MAPK4 consists of a SEG sequence and SPR motif. Thus, MAPK4 can not be phosphorylated by the dual Ser/Thr and Tyr MAPK kinase (MAPKK) [11, 12]. Transcriptomic profiling data provided by The Cancer Genome Atlas (TCGA) [13] show that MAPK4 expression is correlated with the survival rates in patients with lung cancer, bladder cancer and glioma. A recent report demonstrated that MAPK4 activates protein kinase B (AKT) in a phosphatidylinositol 3-hydroxykinase (PI3K)-independent manner and promotes cell proliferation and xenograft tumor growth [14]. After exposure to ionizing radiation, AKT phosphorylation plays a pivotal role in the stimulation of DNA-PKcs and recruitment of AKT1/DNA-PKcs complex as an initiating step of DNA double-strand break (DNA-DSB) repair [15]. A single gene may play different roles depending on the tissue context. In this study, the expression and function of MAPK4 were determined in cells of lung cancer, colon cancer and prostate cancer. Nevertheless, the functions of MAPK4 in radiation and its involvement in diseases, including cervical cancer, requires further investigation.

As an abundant nuclear protein implicated in DNA single-strand break (DNA-SSB) repair, PARP1 plays a vital role in catalyzing poly ADP-ribose formation and maintaining genome integrity [16]. Suppression of PARP1 leads to defective DNA-SSB repair and results in accumulation of DNA-DSB [17]. Given the regulatory roles of MAPK4/AKT and PARP1 in DNA-DSB and DNA-SSB repair, respectively, the possible synergistic effects between MAPK4 deletion and PARP1 inhibition requires further elucidation. Previous study showed that PARP1 hyperactivation leads to therapeutic resistance, and the therapeutic potential of PARP inhibition in combination with cisplatin has shown profound anti-cancer effect in cervical cancer [18]. In addition, high PARylation activity is correlated with sensitivity to olaparib in cervical cancer cells, and represents a biomarker for the identification of patients likely to benefit from PARP1 inhibition [19]. However, the synergistic role of PARP1 inhibition in combination with MAPK4 intervention for cervical cancer therapy is yet to be elucidated.

Here we report that high MAPK4 expression is associated with significantly decreased overall survival rate in cervical cancer patient. Cervical cancer cells were more sensitive to radiation treatment after MAPK4 knockout, as determined by colony formation, immunofluorescence and western blotting. Based on the results from the protein analysis, we found that MAPK4 specifically activated AKT phosphorylation and further affected DNA repair. In addition, MAPK4 knockout enhanced the sensitivity of cervical cancer cells to PARP1 inhibitors, olaparib and veliparib. The synergistic lethality of MAPK4 deletion in combination with PARP1 inhibition was further evaluated in a xenograft mouse model. Together, these results demonstrated that MAPK4 knockout alongside with PARP1 inhibition may improve the therapy of cervical cancer.

## Methods

### Patient tissue samples

Paired cervical cancer tissues (tumor, *n* = 60) and normal adjacent tissues (non-tumor, n = 60) were obtained from the Henan Cancer Hospital. All patients were pathologically and clinically diagnosed as cervical cancer and received no chemotherapy or radiotherapy before section. The collection and experiments were performed under the permission of the Ethics Committee of Henan Cancer Hospital (Approval no.2016HC047). Written informed consents were obtained from all participants.

### Quantitative real-time PCR (qRT-PCR)

First, total RNA was extracted from tissues and cells using the TRIzol reagent (TaKaRa, Otsu, Japan) according to the manufacturerʼs instructions. Total RNA was then converted into complementary DNA (cDNA) using a PrimerScript™ RT Reagent Kit (TaKaRa), following standard procedures. qRT-PCR was performed using a One-Step SYBR PrimeScript RT-PCR Kit (TaKaRa) on a 7500 Real-Time PCR System (Applied Biosystems, Lincoln Centre Drive Foster City, CA). β-Actin was used as a housekeeping gene to normalize the gene expression. The primer sequences for MAPK4 and RAD51 recombinase (RAD51) are listed in Table S1.

### Overall survival analysis

After qRT-PCR analysis, mean MAPK4 mRNA level was calculated and used as a criterion to estimate the expression of MAPK4. If the MAPK4 mRNA level was higher than the mean value, the corresponding patient was identified as “MAPK4 high”, whereas an MAPK4 mRNA level lower than the mean value was denoted as “MAPK4 low”. After surgery combined with radiotherapy or postoperative radiotherapy, follow-up data was collected and the overall survival rate of cervical cancer patients in “MAPK4 high” and “MAPK4 low” group was analyzed using the Kaplan-Meier method.

### Cell culture

In the present study, human cervical cancer cells, SiHa and caSki, were obtained from the ATCC cell lines (Manassas, USA). Cells were recently authenticated by STR profiling. Eagle’s Minimum Essential Medium (EMEM) and RPMI-1640 medium were used to culture SiHa and caSki cells, respectively. Cells were cultured in medium supplied with 10% FBS (Thermo Fisher Scientific) in a humidified atmosphere with 5% CO_2_ at 37 °C.

### Generation of MAPK4 knockout cell lines

SiHa and caSki cells with MAPK4 deletion were established by CRISPR-Cas9 technique as previously reported [14]. Briefly, lentiviral packaging vectors pMD2.G and psPAX2 along with sgRNA-expressing vector, CRISPR-v2 vector, were transfected into HEK293T cells using the Lipo2000 transfection reagent (Thermo Fisher Scientific). The sgRNA sequence for MAPK4 used in study is ACTTCACTGTTCACTTCAGGGAG. Viruses were collected 72 h after transfection and infected SiHa and caSki cells. Then, MAPK4-deleted stable cell lines were selected by puromycin and were continuously monitored by western blotting to avoid potential reversal effects.

### Western blotting

Western blotting was performed as previously reported [20]. Briefly, we collected protein lysates from cells and tissues using RIPA lysis buffer (Sigma-Aldrich, 20 mM Tris pH 7.5, 150 mM NaCl, 1 mM PMSF, 10 mM β-glycerophosphate, 1% Triton X-100, 5 mM EDTA, 0.2 mM Na_3_VO_4_, 2 μg/mL leupeptin, 2 μg/mL pepstatin A). Protein lysates were quantified by a BCA protein assay kit (Thermo Fisher Scientific) and loaded onto the sodium dodecyl sulfate-polyacrylamide gels (SDS-PAGE) for electrophoresis. Next, proteins were transferred onto a PVDF membrane. Membranes were blocked with 5% BSA, and incubated with primary antibodies at 4 °C overnight. After that, HRP (horseradish peroxidase)-conjugated secondary antibodies were used to incubate the membrane. Immunoreactivity was determined by an immobilon ECL western HRP substrate and (Millipore, Billerica, MA, USA) and a chemiluminescence imaging system. β-actin protein level was used as a control. Antibodies used in this study are as follows: MAPK4, phosphorylated-DNA-dependent protein kinase (p-DNA-PK), RAD51, p-AKT T308, p-AKT s473, AKT, AKT1, AKT2, p-AKT and phosphorylated histone H2AX (γH2AX). Western blot bands were quantified by ImageJ and the quantification is shown in histogram. Antibody details are listed in Table S2.

### Irradiation and colony formation assay

Irradiation was performed by X-rays at a dose rate of 5 Gy/min using a linear accelerator (PRIMUS-M, Siemens) for 2 h. After radiation treatment, SiHa or caSki cells were primarily blended into top agar, and the mixture was then added onto base agar. After three weeks, colonies were stained with crystal violet and counted by dissection microscope (Nikon, Tokyo, Japan).

### Immunofluorescence

Cells were seeded and treated with 5 Gy irradiation following cell adhesion. After fixation with 4% paraformaldehyde, SiHa or caSki cells were permeabilized in 0.1% Triton X-100. Then, cells were blocked with 5% BSA and incubated with anti-53BP1 and anti-γH2AX primary antibodies overnight at 4 °C. Cy5-conjugated and fluorescein isothiocyanate (FITC)-conjugated secondary antibodies were used. To visualize nuclear DNA, cell nuclei were stained with DAPI (4′,6-diamidino-2-phenylindole, Sigma-Aldrich, St. Louis, MO, USA). Cells were observed and photographed using a confocal microscope (Nikon).

### Cell transfection and cell treatment

SiHa or caSki cells were transfected with MAPK4 expressing plasmid (MAPK4), shRNA for MAPK4 (sh-MAPK), siRNA for AKT1 and AKT2 (si-AKT1, si-AKT2), plasmid with constitutively activated AKT (CA-AKT) or the corresponding controls (Ctrl or si-NC) using Lipofectamine 2000 (Thermo Fisher Scientific). Three shRNAs for MAPK4, three siRNAs for AKT1 and AKT2 were tested. The siRNA sequences are listed in Table S3. Oligos with highest efficiency were used for subsequent experiments. To construct the MAPK4-overexpressing vector, a One Step Cloning Kit (Vazyme Biotech, Nanjing) was used according to the manufacturer’s instructions. The coding sequence (CDS) of MAPK4 was amplified using a human genomic DNA extracted from SiHa cells and then inserted into the PCI plasmid (Promega, Madison, WI, USA). Primers sequences for cloning MAPK4 CDS into the expressing vector are listed in Table S4. CA-AKT plasmid was purchased from Addgene (catalog no. plasmid #14751, Watertown, MA).

### Synergistic effect of MAPK4 deletion with PARP1 inhibitors

After cells were incubated with different concentrations of PARP1 inhibitors, olaparib or veliparib, cell viablity was determined by a cell counting kit-8 (CCK-8, Dojindo, Tokyo, Japan). A microplate spectrometer (Thermo Fisher Scientific) was used to determine the absorbance (OD = 450 nm). Next, the 50% inhibitory concentration (IC50) was quantified using the sigmoidal dose-response function of GraphPad Prism. In addition, after knocking out MAPK4, SiHa or caSki cells were over-expressed with MAPK4 or CA-AKT. Accordingly, the IC50 was quantified.

### Xenograft mouse model

Animal experiments were performed according to the Guidelines for the Care and Use of Laboratory Animals published by the National Institutes of Health [21] and approved by the Ethics Committee of Henan Cancer Hospital. Thymic BALB/c nude mice (female, four-six weeks, 16-18 g) were housed in a pathogen-free facility and used for xenograft studies. SiHa or caSki cells with MAPK4 deletion (1 × 10^6^) and the control cells were subcutaneously injected into the right flanks of mice. When the tumors reached a diameter of 4–5 mm, mice were randomly assigned to different groups (6 mice/group). Tumors were monitored and treated with 5 Gy irradiation every 2 days after the injection. The length (L) and width (W) of tumors were determined every week. Then, the volume of each tumor was calculated using the formula V = 1/2LW^2^. At 4 weeks post-implantation, tumors were dissected from the mice. Protein expression of p-AKT, AKT, p-DNA-PK, MAPK and γH2AX in tumor tissues was analyzed in each group established by caSki cells. To study the synergistic effect of MAPK4 deletion with PARP1 inhibitors in vivo, olaparib (100 mg/kg) was administered by oral gavage once daily, in addition to irradiation treatment. Tumor volume (mm^3^) of each group median was graphed over time to monitor tumor growth. 4 weeks later, tumors were harvested and photographed.

### Statistical analysis

The statistical data were obtained from three independent experiments and fit the normal distribution. Data are presented as the mean ± standard error of the mean (SEM). The investigator was blinded to the group allocation during the experiment. Statistical differences were analyzed using the Student *t* test for two groups and ANOVA for multiple groups. Variation within each group of data was estimated, and the variance between groups was statistically compared. *P* < 0.05 was considered as significant.

## Results

### MAPK4 level is associated with the survival of cervical cancer patients

**Fig. 1:**
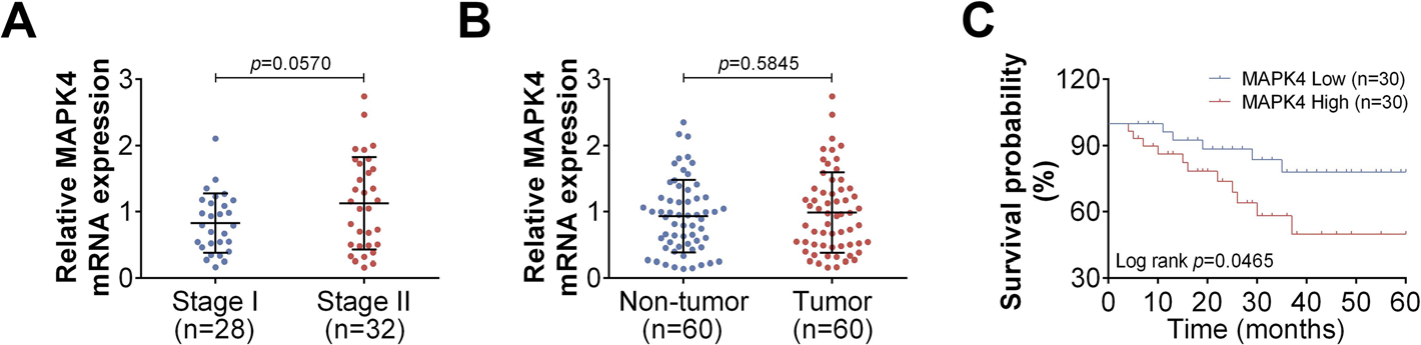
MAPK4 mRNA level is associated with the overall survival of cervical cancer patients. **a** Relative MAPK4 mRNA levels in tissues of cervical cancer patients at stage I (*n* = 28) or stage II (*n* = 32), as determined using qRT-PCR. **b** Relative MAPK4 mRNA levels in cervical cancer tissues (tumor, *n* = 60) and normal adjacent tissues (non-tumor, n = 60), as determined using qRT-PCR. **c** Overall survival curve analysis of 60 cervical cancer patients using Kaplan-Meier method, stratified by MAPK4 mRNA expression. Data are expressed as the mean ± SEM

To study the correlation between MAPK4 level and cervical cancer development, we determined the MAPK4 mRNA levels in cervical cancer tissues of different disease stages (*n* = 60). There was no significant difference in the relative MAPK4 mRNA level in stage II cervical cancer patients (*n* = 32) and stage I cervical cancer patients (*n* = 28, Fig. 1a). Similar results were observed in tumor tissues (cervical cancer tissues, *n* = 60) and non-tumor tissues (corresponding normal adjacent tumor tissues, n = 60, Fig. 1b). However, when analyzing the relationships between MAPK4 mRNA level and the survival of cervical cancer patients after chemoradiotherapy, it was found that the survival rate of cervical cancer patients in “MAPK4 High” group was lower than that of patients in “MAPK4 Low” group (Fig. 1c). Because MAPK4 expression is associated with patient survival, these result suggest that MAPK4 may serve as a potential synergetic therapeutic target with PARP1 inhibitors in radiotherapy of cervical cancer. These findings led us to examine whether MAPK4 deletion can improve the effect of chemoradiotherapy for cervical cancer.

### MAPK4 knockout increases the sensitivity of cervical cancer cells to radiation

**Fig. 2:**
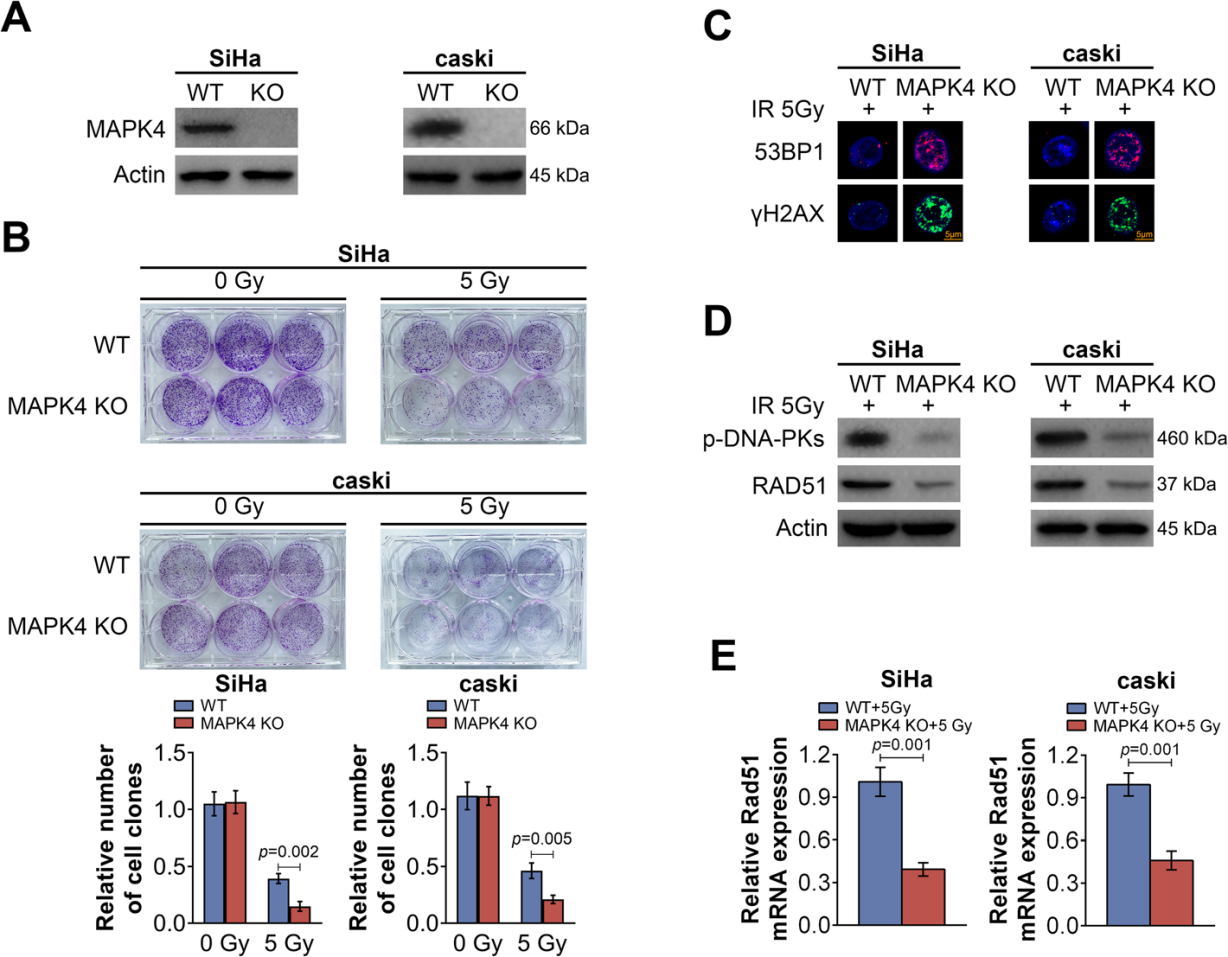
MAPK4 knockout increases the sensitivity of cervical cancer cells to radiation. **a** Relative protein levels of MAPK4 in SiHa or caSki WT cells and MAPK4 KO cells after radiation treatment, as determined using western blotting. **b** Colony formation analysis of SiHa or caSki WT cells and MAPK4 KO cells after radiation treatment. The number of cell clones was quantified and shown as histogram. **c** The 53BP1 and γH2AX levels in SiHa or caSki WT cells and MAPK4 KO cells after radiation treatment, as determined using immunofluorescence. Representative images of three independent experiments are shown (Scale bar, 5 μm). **d** Relative protein levels of p-DNA-PK and RAD51 in SiHa or caSki WT cells and MAPK4 KO cells after radiation treatment, as determined using western blotting. **e** Relative RAD51 mRNA levels in SiHa or caSki WT cells and MAPK4 KO cells after radiation treatment, as determined using qRT-PCR. Data are expressed as the mean ± SEM

To study the effect of MAPK4 knockout on the sensitivity of cervical cancer cells to radiation, MAPK4-deleted SiHa or caSki stable cell lines were established by CRISPR-Cas9 technique. The western blotting results did not detect MAPK4 expression in the cells with MAPK4 knockout (MAPK4 KO), confirming the successful establishment of MAPK4 knockout cell line (Fig. 2a). Colony formation assay was then performed after radiation treatment. The results showed that MAPK4 knockout itself did not significantly affect the colony formation of cervical cancer cells (Fig. 2b). With radiation treatment at 5 Gy, the colony number of MAPK4 knockout cells was markedly reduced compared to that of wild type (WT) cells. After MAPK4-knockout cervical cancer cells were treated with 5 Gy irradiation, the markers for DNA double-chain breakage were detected by immunofluorescence. After MAPK4 was knocked out, 53BP1 and γH2AX levels were significantly increased, indicating more severe DNA damage (Fig. 2c). Western blotting was used to assess the levels of proteins associated with DNA repair. p-DNA-PK and RAD51 were significantly decreased following MAPK4 knockout, indicating that DNA repair was weakened (Fig. 2d). This was further confirmed by qRT-PCR results, which showed that the transcript levels of RAD51 in SiHa and caSki cells were significantly decreased (Fig. 2e). Together, these data suggest that MAPK4 knockout could down-regulate cell proliferation, induce DNA damage and attenuate DNA repair in cervical cancer cells after irradiation treatment.

### MAPK4 activates AKT phosphorylation in cervical cancer cells

**Fig. 3:**
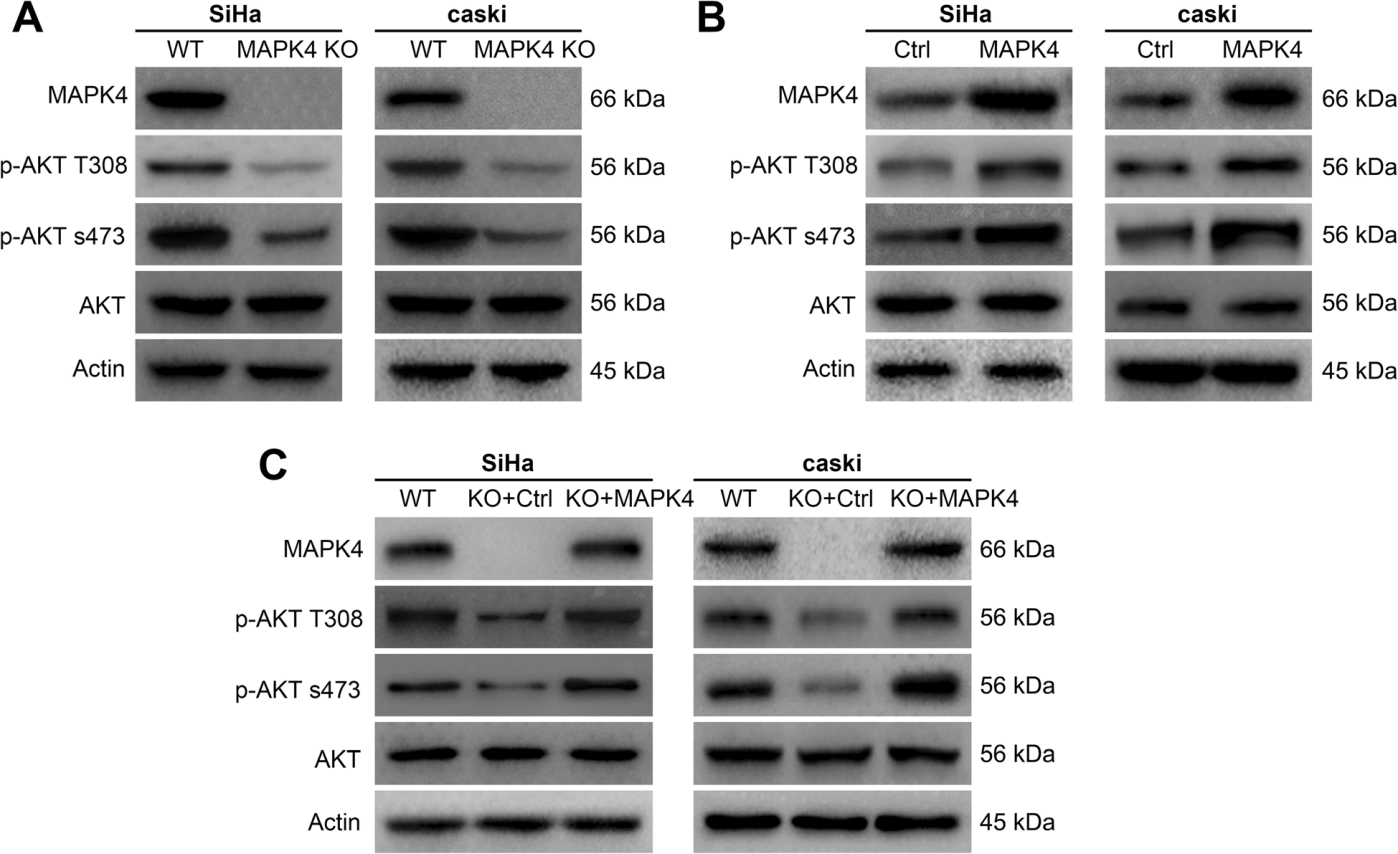
MAPK4 activates AKT phosphorylation in cervical cancer cells. **a** Relative protein levels of MAPK4, p-AKT T308, p-AKT s473, AKT in SiHa or caSki WT cells and MAPK4 KO cells, as determined using western blotting. **b** Relative protein levels of MAPK4, p-AKT T308, p-AKT s473, AKT in SiHa or caSki Ctrl cells and MAPK4 over-expressing cells, as determined using western blotting. **c** Relative protein levels of MAPK4, p-AKT T308, p-AKT s473, AKT in SiHa or caSki WT cells, MAPK4 KO cells and MAPK4 over-expressing cells, as determined using western blotting. Representative images of three independent experiments are shown

As shown in Fig. 3a, western blotting was used to determine the protein levels of p-AKT T308, p-AKT s473 and AKT in MAPK4-knockout SiHa or caSki cells. The absence of MAPK4 led to a decrease in the phosphorylation levels of AKT at T308 and s473 sites, but the total amount of AKT remained unaltered, suggesting that MAPK4 knockdown may inhibit AKT phosphorylation. In addition, the phosphorylation levels of AKT were determined in MAPK4-overexpressing cervical cancer cells, which demonstrated an increase in phosphorylated AKT, but had no significant change in the levels of total AKT. This suggests that MAPK4 overexpression can promote AKT phosphorylation (Fig. 3b). Next, we further explored whether dysregulated AKT phosphorylation was directly caused by MAPK4 knockout or over-expression. We re-expressed MAPK4 using specific expressing plasmids in MAPK4 knockout cervical cancer cells, and examined the phosphorylation levels of AKT. Our results showed a decrease in phosphorylated AKT following MAPK4 knockout, which was restored by over-expressing MAPK4. There was no significant change in total AKT (Fig. 3c). Thus, these data further confirmed that MAPK4 could promote AKT phosphorylation.

### MAPK4 affects DNA repair through regulating AKT phosphorylation

**Fig. 4:**
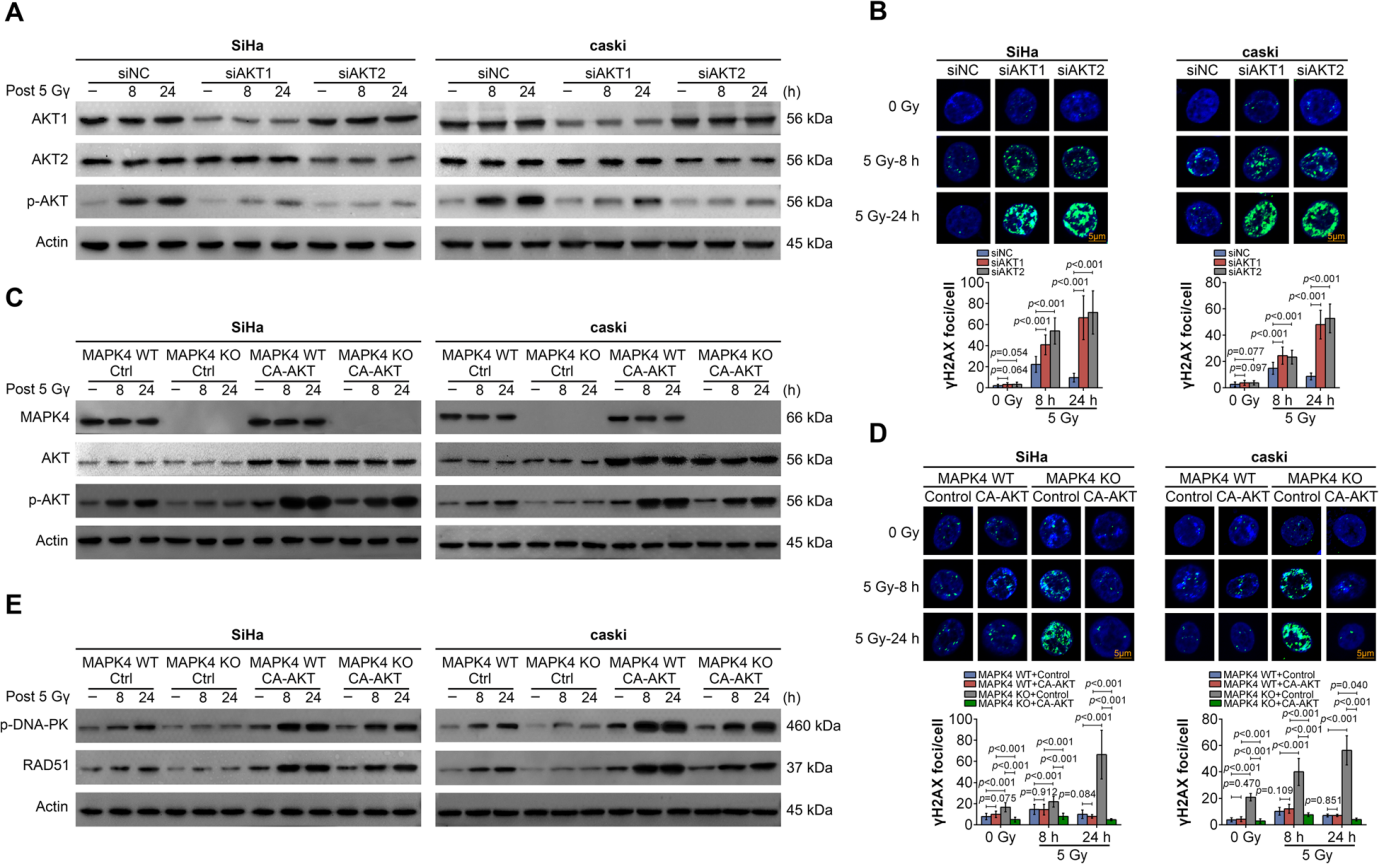
MAPK4 affects the sensitivity of cervical cancer cells to radiation by regulating AKT phosphorylation. **a** Relative protein levels of AKT1, AKT2 and p-AKT in SiHa or caSki cells after transfecting siNC, siRNAs for AKT1 or AKT2 at 0 h, 8 h and 24 h, as determined using western blotting. **b** The γH2AX levels in SiHa or caSki cells after transfecting siNC, siAKT1 or siAKT2 at 0 h, 8 h and 24 h, as determined using immunofluorescence. Fluorescence intensity was quantified and shown as histogram (Scale bar, 5 μm). **c** Relative protein levels of MAPK4, AKT and p-AKT in SiHa or caSki MAPK4 WT cells and MAPK4 KO cells after transfecting Ctrl or CA-AKT at 0 h, 8 h and 24 h, as determined using western blotting. **d** The γH2AX levels in SiHa or caSki cells after transfecting siNC, siAKT1 or siAKT2 at 0 h, 8 h and 24 h, as determined using immunofluorescence. Fluorescence intensity was quantified and shown as histogram (Scale bar, 5 μm). **e** Relative protein levels of p-DNA-PK and RAD51 in SiHa or caSki MAPK4 WT cells and MAPK4 KO cells after transfecting Ctrl or CA-AKT at 0 h, 8 h and 24 h, as determined using western blotting. Representative images of three independent experiments were shown. Data are expressed as the mean ± SEM

As mentioned above, we found that MAPK4 could affect DNA repair and the phosphorylation of AKT. Next, we sought to determine whether MAPK4 affects DNA repair through regulating AKT phosphorylation. Cervical cancer cells were transfected with AKT1 or AKT2 siRNA and subjected to irradiation treatment. The western blotting results showed that after AKT1 and AKT2 were knocked down, AKT1, AKT2 and p-AKT levels were decreased. Irradiation treatment led to the up-regulation of p-AKT. However, when AKT1 or AKT2 was silenced, the effect of irradiation treatment on the p-AKT level was reduced (Fig. 4a). Accordingly, immunofluorescence results showed that γH2AX levels were increased after AKT1 or AKT2 was knocked down, indicating that AKT reduction could promote DNA damage (Fig. 4b). In addition, p-AKT and γH2AX levels were determined in MAPK4-knockout cervical cancer cells that constitutively expressed AKT. The over-expression of CA-AKT led to an increased p-AKT level (Fig. 4c) and a reduction in γH2AX (Fig. 4d) in MAPK4 KO cells with irradiation treatment. Besides, the levels of DNA repair related proteins, p-DNA-PK and RAD51, were determined in MAPK4 KO cells with irradiation treatment and CA-AKT over-expression. After over-expressing CA-AKT, p-DNA-PK and RAD51 levels were significantly up-regulated, suggesting that CA-AKT could promote DNA repair (Fig. 4e). These data above revealed that MAPK4 affected DNA repair through regulating AKT phosphorylation in cervical cancer cells.

### MAPK4 knockout enhances the sensitivity of cervical cancer cells to PARP1 inhibitors

**Fig. 5:**
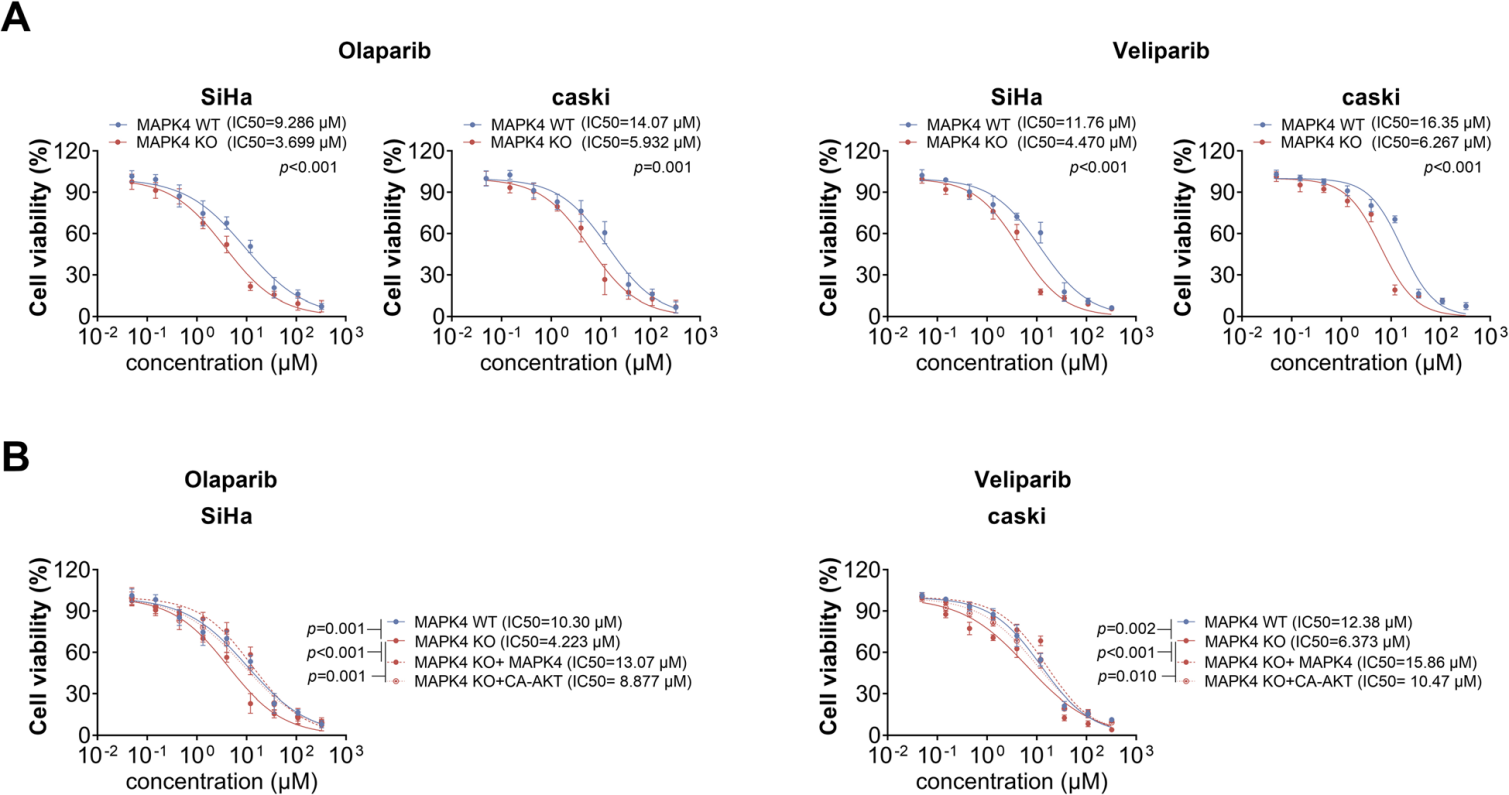
MAPK4 knockout affects the sensitivity of cervical cancer cells to PARP1 inhibitors. **a** Relative cell viability of SiHa or caSki MAPK4 WT cells and MAPK4 KO cells after treatment with different concentrations of olaparib or veliparib, as determined by CCK-8. IC50 was calculated using the sigmoidal dose-response function of GraphPad Prism. **b** Relative cell viability of SiHa MAPK4 WT cells, MAPK4 KO cells, MAPK4 KO with MAPK4 over-expression cells (MAPK4 KO + MAPK4 cells) and MAPK4 KO with CA-AKT over-expression cells (MAPK4 KO + CA-AKT cells), as determined by CCK-8. IC50 was calculated using the sigmoidal dose-response function of GraphPad Prism. Data are expressed as the mean ± SEM

Given that MAPK4 knockout led to defective repair of DNA double-strand breaks, we next speculated that MAPK4 knockout and PARP1 inhibition may exert synergistic effects, leading to dysfunctional DNA-SSB repair. Thus, we then investigated the effect of MAPK4 knockout on the sensitivity of cervical cancer cells to PARP1 inhibitors, olaparib or veliparib. As shown in Fig. 5a, the IC50 value of olaparib in SiHa or caSki cells was significantly decreased after MAPK4 knockout. Similar results were observed in veliparib treated-SiHa or caSki cells. Furthermore, the IC50 of PARP1 inhibitors was detected by re-expressing MAPK4 or over-expressing CA-AKT in MAPK4 knockout cervical cancer cells. As shown in Fig. 5b, decreased-IC50 in MAPK4 KO cells could be restored after re-expressing MAPK4 or over-expressing CA-AKT. These findings suggest that MAPK4 affects the sensitivity of cervical cancer cells to PARP1 inhibitors, through activating AKT phosphorylation.

### MAPK4 knockout enhances the sensitivity of cervical cancer to radiation and PARP1 inhibitors in vivo

**Fig. 6:**
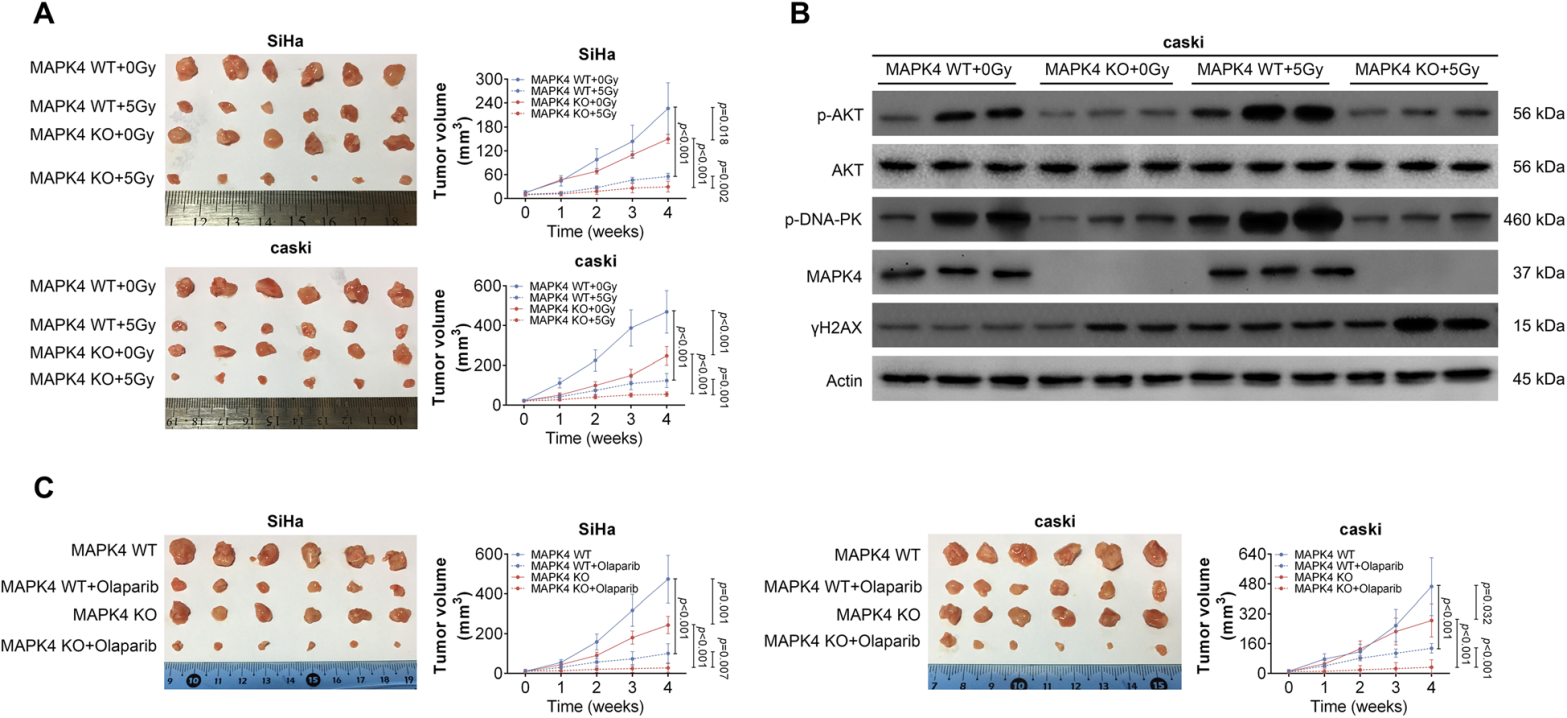
MAPK4 knockout enhances the sensitivity of cervical cancer cells to radiation treatment and PARP1 inhibitors. **a** 4 weeks post-implantation, image of corresponding xenograft tumors derived from subcutaneous implantation of SiHa or caSki MAPK4 WT cells with no radiation treatment (MAPK4 WT + 0Gy), MAPK4 WT cells with radiation treatment (MAPK4 WT + 5Gy), MAPK4 KO cells with no radiation treatment (MAPK4 KO + 0Gy), MAPK4 KO cells with radiation treatment (MAPK4 KO + 5Gy). Volumes of the xenograft tumors were measured weekly and shown as line diagrams. **b** Relative protein levels of p-AKT, AKT, p-DNA-PK, MAPK4 and γH2AXin corresponding xenograft tumors derived from subcutaneous implantation of SiHa or caSki MAPK4 WT cells with no radiation treatment (MAPK4 WT + 0Gy), MAPK4 WT cells with radiation treatment (MAPK4 WT + 5Gy), MAPK4 KO cells with no radiation treatment (MAPK4 KO + 0Gy), MAPK4 KO cells with radiation treatment (MAPK4 KO + 5Gy), as determined by western blotting. **c** 4 weeks post-implantation, image of corresponding xenograft tumors derived from subcutaneous implantation of SiHa or caSki MAPK4 WT cells (MAPK4 WT), MAPK4 WT cells with olaparib treatment (MAPK4 WT + Olaparib), MAPK4 KO cells (MAPK4 KO), MAPK4 KO cells with olaparib treatment (MAPK4 KO + Olaparib). Volumes of the xenograft tumors were measured weekly and shown as line diagrams. Data are expressed as the mean ± SEM

Given that MAPK4 deletion enhanced the sensitivity of cervical cancer cells to radiation and PARP1 inhibitors, we next investigated the effect of MAPK4 deletion in vivo. To construct a xenograft tumor mouse model, MAPK4-deleted stable SiHa and caSki cell lines were subcutaneously injected into nude mice. Subsequently, irradiation treatment was performed once a day at 2 Gy, and the tumor growth curve was detected. As shown in Fig. 6a, MAPK4 knockout inhibited tumor growth to a certain extent. This observation was more evident after irradiation, in which MAPK4 knockout caused a more significant inhibition on tumor development. Furthermore, p-AKT, AKT, p-DNA-PK, MAPK4 and γH2AX protein levels were determined in xenograft tumors (Fig. 6b). MAPK4 knockout, especially after irradiation, led to down-regulated p-AKT and pDNA-PK and up-regulated γH2AX. In addition, after MAPK4 KO cells were subcutaneously injected into the mice, gavage of PARP1 inhibitor olaparib was performed irradiation treatment once a day. The results suggest a synergistic inhibitory effect of MAPK4 knockout and PARP1 inhibitor on tumor growth in vivo (Fig. 6c). Together, these findings demonstrated that MAPK4 knockout could inhibit DNA repair and improve the sensitivity of cervical cancer to irradiation treatment and PARP1 inhibitors in vivo.

## Discussion

In this study, we have not only demonstrated the suppressive role of MAPK4 knockout on AKT phosphorylation, but also identified the relationship between MAPK4 knockout and enhanced sensitivity of cervical cancer to radiation treatment and PARP1 inhibitors in cells and mouse models. Although intensity-modulated radiotherapy (IMRT) has been adopted to reduce gastrointestinal toxicity and increase the radiotherapy dose, treatment tolerance and severe side effects are dose-limiting factors. Thus, novel developments aim to improve radiotherapy for cervical cancer [22]. It was demonstrated that concomitant radiochemotherapy could improve disease-free and overall survival compared to radiotherapy alone in early cervical cancer [23]. For example, methyl jasmonate (MJ), a newly identified cytotoxic agent, when effectively incorporated with X-ray irradiation, can significantly reduce the radiation doses required to inhibit cell survival of cervical cancer cells [24]. Additionally, the combination of Aloe-emodin (AE), an *Aloe vera* leaf exudate and radiation induce apoptosis and further improve Alkaline phosphatase (ALP) activity compared with treatment with AE or radiation alone [25]. Our data in this study demonstrated that MAPK4 knockout could enhance the sensitivity of cervical cancer cells to radiation treatment both in vitro and in vivo, suggesting that targeting MAPK4 may be a promising radiosensitizer.

As an atypical member of the mitogen-activated protein (MAP) kinase family, MAPK4 knockout mice are viable and fertile and exhibit no gross morphological or physiological anomalies. However, MAPK4-deficient mice manifest depression-like behavior in forced-swimming tests, indicating that the MAPK4 has acquired specialized functions through evolutionary diversification [26]. So far, little is known about the physiological function of MAPK4 and its involvement in diseases, including cancer. Although gene expression profiling data provided by The Cancer Genome Atlas (TCGA) show that MAPK4 expression is correlated with the survival rates in patients with lung cancer, bladder cancer and glioma, its functions and mechanism of actions in lung cancer and colon cancer were recently identified [13]. Wang et al. demonstrated that over-expression of MAPK4 leads to oncogenic effects, and MAPK4 inhibition suppresses cell proliferation and xenograft tumor growth. Mechanistically, MAPK4 activates the phosphorylation of AKT at threonine 308 and serine 473 [14]. Our data in this study demonstrated that cervical cancer patients with high MAPK4 expression had lower survival probability and MAPK4 deletion blocked AKT phosphorylation in cervical cancer cells. AKT phosphorylation has previously been described to cooperate with DNA-PKcs and was involved in DNA damage repair. AKT1 is a regulatory component in the homologous recombination repair of DNA-DSB in a Rad51-dependent manner in non-small cell lung cancer cells [27]. Single knockdown of Akt1 and Akt2 leads to a decrease in Rad51 foci formation and significantly reduces Rad51 protein level in colon cancer cells [28]. Moreover, Akt1-T308A/S473A-expressing cells are characterized by increased radiosensitivity compared to Akt1-WT (wild type)-expressing cells in long-term colony formation assays [29]. Dual targeting of mTORC1 and AKT1 inhibits DNA-DSB repair, leading to radiosensitization of solid tumor cells [30]. We found that MAPK4-knockout cervical cancer cells showed lower AKT phosphorylation level, and had heightened sensitivity to radiation treatment and PARP1 inhibitors.

In regard to the upstream regulation of MAPK4, two miRNAs have been reported to specifically target MAPK4. Over-expression of miR-767-5p functions as a tumor drive through targeting MAPK4 in multiple myeloma [31]. miR-127 was found to target both MAPK4 and HOXC6, and promotes cell proliferation and decreases differentiation in porcine [32]. These indicate that the expression and functions of MAPK4 may vary depending on the cellular context. To date, the regulatory mechanism of MAPK4 in cervical cancer remains unclear, and whether or not miR-767-5p and miR-127 could target MAPK4 and other potential transcriptional regulatory factors will require further investigation.

Because radiotherapy alone or concurrent chemoradiation fail to control advanced cervical cancer, surgery, chemotherapy or targeted therapy have been used in combination to improve the radiotherapy and minimize the side effects. Studies are currently being carried out to investigate potential suppressors of survival pathways and promoters of apoptotic pathways as novel chemotherapy approaches for the treatment of cervical cancer [33]. An association between poly ADP-ribose polymerase 1 (PARP1) Val762Ala polymorphism (rs1136410) and cancer therapy response has been identified, and PARP1 genotypes have been proposed to be an independent prognostic factor in cervical cancer [34]. In addition, it has been shown that PARP-1 inhibition may augment cisplatin cytotoxicity in cervical cancer cells by diminishing DNA repair and suppressing β-catenin signaling pathway [35]. miR-7-5p was demonstrated to negatively regulate PARP-1 protein and gene expression in cisplatin-resistant cervical cancer cells, and facilitates DNA repair and maintain cell survival [36]. However, little is known about the therapeutic efficacy of PARP inhibitors in the treatment of cervical cancer, either as a single agent or in combination with MAPK4 knockout. PARP1 inhibition along with superoxide dismutase 1 (SOD1) inhibition could promote the synthetic lethal killing of RAD54B-deficient colorectal cancer cells [37]. Previous study has also shown that simultaneous treatment with PARP and RAD52 inhibitors exerts dual synthetic lethality in BRCA-deficient tumors [38]. Currently, PARP inhibitors are under clinical trials for BRCA1/BRCA2-deficient breast cancer and ovarian cancer by the approach of synthetic lethal [39, 40]. Our results demonstrate for the first time that addition of PARP inhibitor may improve therapeutic outcome of MAPK4-deficient cervical cancer treated with radiation.

## Conclusions

Altogether, our data revealed that cervical cancer patients with high MAPK4 mRNA expression have lower survival rate. After radiation treatment, the colony number of MAPK4 knockout cells was markedly reduced, and the markers for DNA double-chain breakage were significantly up-regulated. In addition, MAPK4 knockout reduced the phosphorylation of AKT, whereas its over-expression exerted opposite effects. In MAPK4 KO cells with irradiation treatment, inhibition of AKT phosphorylation promoted DNA double-chain breakage, and constitutively activated AKT (CA-AKT) increased the levels of p-AKT and DNA repair related proteins, p-DNA-PK and RAD51. MAPK4 is further found to affect the sensitivity of cervical cancer cells to PARP1 inhibitors by activating AKT phosphorylation. Moreover, MAPK4 knockout enhanced the sensitivity of cervical cancer to radiation and PARP1 inhibitors in mouse xenograft models. Collectively, our data suggest that combined application of MAPK4 knockout and radiation treatment or PARP1 inhibition can be used as therapeutic strategy for advanced cervical carcinoma.

## Supplementary information

**Additional file 1: Table S1.** The primers for qRT-PCR assay. **Table S2.** Antibodies used in this study. **Table S3.** The sequences for siRNAs. **Table S4.** The primers for MAPK4 expressing plasmid construct.

**Additional file 2.** The prognosis of survival.

## Data Availability

All data generated or analyzed during this study are included in this published article.

## Abbreviations

MAPK4: Mitogen-activated protein kinase 4
IC50: Inhibitory concentration
CA-AKT: Constitutively activated AKT
p-AKT: Phosphorylated-AKT
p-DNA-PK: Phosphorylated-DNA-dependent protein kinase
RAD51: RAD51 recombinase
PARP1: Poly ADP-ribose polymerase 1
HPV: Hhuman papillomavirus
NCCN: National Comprehensive Cancer Network
MAPKK: MAPK kinase
TCGA: The Cancer Genome Atlas
AKT: Activates protein kinase B
PI3K: Phosphatidylinositol 3-hydroxykinase
PARP1: Polymerase 1
