# Supplementary material for: MAPK4 deletion enhances radiation effects and triggers synergistic lethality with simultaneous PARP1 inhibition in cervical cancer
Source: J Exp Clin Cancer Res. 2020 Jul 25;39:143. doi: 10.1186/s13046-020-01644-5 (PMC7382858; doi:10.1186/s13046-020-01644-5)
Supplement: Supplementary file 1 — Additional file 1: Table S1. The primers for qRT-PCR assay. Table S2. Antibodies used in this study. Table S3. The sequences for siRNAs. Table S4. The primers for MAPK4 expressing plasmid construct. [file 13046_2020_1644_MOESM1_ESM.doc]

**Supplementary Tables**

**Table S1** The primers for qRT-PCR assay.

| **Primer Name** | **Primer Sequence (5’-3’)** |
| --- | --- |
| β-actin RT primer | random primer |
| β-actin Reverse primer | TGATCTTCATTGTGCTGGGTG |
| β-actin Forward primer | ACATCCGCAAAGACCTGTAC |
| MAPK4 RT primer | random primer |
| MAPK4 Reverse primer | GAGTGGGAGTGTTGGAAGAAG |
| MAPK4 Forward primer | TTACTAAAGGCAAACGGTGAA |
| miR-1236-3p RT primer | random primer |
| miR-1236-3p Reverse primer | TCGATCTCATCCTCAATGCG |
| miR-1236-3p Forward primer | ATCCTGACCTTTAACCCCATG |

**Table S2** Antibodies used in this study.

| **Antibody name** | **Corporation name** | **Catalog** | **Source** | **Poly/monoclonal** |
| --- | --- | --- | --- | --- |
| MAPK4 | Thermo Fisher Scientific | MA5-26208 | mouse | monoclonal |
| p-DNA-PK | Cell Signaling Technology | 68716 | rabbit | monoclonal |
| RAD51 | Cell Signaling Technology | 8875 | rabbit | monoclonal |
| p-AKT T308 | Cell Signaling Technology | 13038 | rabbit | monoclonal |
| p-AKT s473 | Cell Signaling Technology | 4060 | rabbit | monoclonal |
| AKT | Cell Signaling Technology | 2920 | mouse | monoclonal |
| AKT1 | Cell Signaling Technology | 75692 | rabbit | monoclonal |
| AKT2 | Cell Signaling Technology | 3063 | rabbit | monoclonal |
| p-AKT | Cell Signaling Technology | 9611 | rabbit | monoclonal |
| H2AX | Cell Signaling Technology | 7631 | rabbit | monoclonal |
| GAPDH | Thermo Fisher Scientific | AM430 | mouse | monoclonal |
| Goat anti-rabbit IgG | Thermo Fisher Scientific | 31466 | goat | monoclonal |
| Goat anti-mouse IgG | Thermo Fisher Scientific | 31431 | goat | monoclonal |

**Table S3** The sequences for siRNAs.

| **SiRNA Name** | **Sequence (5’-3’)** |
| --- | --- |
| AKT1 siRNA1 | CCTCAAGAATGATGGCACCTTCATT |
| AKT1 siRNA2 | CACTGTCATCGAACGCACCTTCCAT |
| AKT1 siRNA3 | GCGTGACCATGAACGAGTTTGAGTA |
| AKT2 siRNA1 | GGGCTAAAGTGACCATGAA |
| AKT2 siRNA2 | GGCTAAAGTGACCATGAAT |
| AKT2 siRNA3 | GCAAGGGAACCTTTGGCAA |

**Table S4** The primers for MAPK4 expressing plasmid construct.

| **Primer Name** | **Primer Sequence (5’-3’)** |
| --- | --- |
| MAPK4-PCI Reverse primer | CGGGTCGACTCTAGAGGTACCTCACCACCTTTCTTTGGAGAAGG |
| MAPK4-PCI Forward primer | CGAGAATTCACGCGTGGTACCATGTGGGCCGCCGGCTGC |
