# Supplementary material for: MAPK4 deletion enhances radiation effects and triggers synergistic lethality with simultaneous PARP1 inhibition in cervical cancer
Source: J Exp Clin Cancer Res. 2020 Jul 25;39:143. doi: 10.1186/s13046-020-01644-5 (PMC7382858; doi:10.1186/s13046-020-01644-5)
Supplement: Supplementary file 2 — Additional file 2. The prognosis of survival. [file 13046_2020_1644_MOESM2_ESM.docx]

| (0 represents low MAPK4 expression, 1 represents high MAPK4 expression) | lifetime | Event（0 is death and 1 is survival） | |
| --- | --- | --- | --- |
| 0 | 6 | 0 |  |
| 0 | 8 | 0 |  |
| 0 | 9 | 0 |  |
| 0 | 11 | 1 |  |
| 0 | 13 | 1 |  |
| 0 | 16 | 0 |  |
| 0 | 18 | 0 |  |
| 0 | 19 | 1 |  |
| 0 | 21 | 0 |  |
| 0 | 23 | 0 |  |
| 0 | 25 | 0 |  |
| 0 | 27 | 0 |  |
| 0 | 29 | 1 |  |
| 0 | 31 | 0 |  |
| 0 | 33 | 0 |  |
| 0 | 35 | 1 |  |
| 0 | 36 | 0 |  |
| 0 | 38 | 0 |  |
| 0 | 40 | 0 |  |
| 0 | 42 | 0 |  |
| 0 | 44 | 0 |  |
| 0 | 46 | 0 |  |
| 0 | 48 | 0 |  |
| 0 | 49 | 0 |  |
| 0 | 51 | 0 |  |
| 0 | 54 | 0 |  |
| 0 | 55 | 0 |  |
| 0 | 57 | 0 |  |
| 0 | 59 | 0 |  |
| 0 | 60 | 0 |  |
| 1 | 4 |  | 1 |
| 1 | 5 |  | 1 |
| 1 | 6 |  | 0 |
| 1 | 7 |  | 1 |
| 1 | 9 |  | 0 |
| 1 | 10 |  | 1 |
| 1 | 12 |  | 0 |
| 1 | 13 |  | 0 |
| 1 | 15 |  | 1 |
| 1 | 16 |  | 1 |
| 1 | 18 |  | 0 |
| 1 | 19 |  | 0 |
| 1 | 20 |  | 0 |
| 1 | 22 |  | 1 |
| 1 | 23 |  | 0 |
| 1 | 25 |  | 1 |
| 1 | 26 |  | 1 |
| 1 | 28 |  | 0 |
| 1 | 29 |  | 0 |
| 1 | 30 |  | 1 |
| 1 | 31 |  | 0 |
| 1 | 33 |  | 0 |
| 1 | 36 |  | 0 |
| 1 | 37 |  | 1 |
| 1 | 38 |  | 0 |
| 1 | 43 |  | 0 |
| 1 | 46 |  | 0 |
| 1 | 49 |  | 0 |
| 1 | 55 |  | 0 |
| 1 | 60 |  | 0 |
